# Vaccination against Bm86 Homologues in Rabbits Does Not Impair *Ixodes ricinus* Feeding or Oviposition

**DOI:** 10.1371/journal.pone.0123495

**Published:** 2015-04-28

**Authors:** Jeroen Coumou, Alex Wagemakers, Jos J. Trentelman, Ard M. Nijhof, Joppe W. Hovius

**Affiliations:** 1 Center for Experimental and Molecular Medicine, Academic Medical Center Amsterdam, University of Amsterdam, Amsterdam, The Netherlands; 2 Institute for Parasitology and Tropical Veterinary Medicine, Freie Universität Berlin, Berlin, Germany; 3 Amsterdam Multidisciplinary Lyme borreliosis Center, Academic Medical Center Amsterdam, University of Amsterdam, Amsterdam, The Netherlands; 4 Department of Infectious Diseases, Academic Medical Center Amsterdam, University of Amsterdam, Amsterdam, The Netherlands; University of Kentucky College of Medicine, UNITED STATES

## Abstract

Human tick-borne diseases that are transmitted by *Ixodes ricinus*, such as Lyme borreliosis and tick borne encephalitis, are on the rise in Europe. Diminishing *I*. *ricinus* populations in nature can reduce tick exposure to humans, and one way to do so is by developing an anti-vector vaccine against tick antigens. Currently, there is only one anti-vector vaccine available against ticks, which is a veterinary vaccine based on the tick antigen Bm86 in the gut of *Rhipicephalus microplus*. Bm86 vaccine formulations cause a reduction in the number of *Rhipicephalus microplus* ticks that successfully feed, i.e. lower engorgement weights and a decrease in the number of oviposited eggs. Furthermore, Bm86 vaccines reduce transmission of bovine Babesia *spp*. Previously two conserved Bm86 homologues in *I*. *ricinus* ticks, designated as Ir86-1 and Ir86-2, were described. Here we investigated the effect of a vaccine against recombinant Ir86-1, Ir86-2 or a combination of both on *Ixodes ricinus* feeding. Recombinant *Ixodes ricinus* Bm86 homologues were expressed in a *Drosophila* expression system and rabbits were immunized with rIr86-1, rIr86-2, a combination of both or ovalbumin as a control. Each animal was infested with 50 female adults and 50 male adults *Ixodes ricinus* and tick mortality, engorgement weights and egg mass were analyzed. Although serum IgG titers against rIr86 proteins were elicited, no effect was found on tick feeding between the rIr86 vaccinated animals and ovalbumin vaccinated animals. We conclude that vaccination against Bm86 homologues in *Ixodes ricinus* is not an effective approach to control *Ixodes ricinus* populations, despite the clear effects of Bm86 vaccination against *Rhipicephalus microplus*.

## Introduction

During a blood meal, *Ixodes ricinus* ticks can transmit several pathogens, including *B*. *burgdorferi spp*. and tick-borne encephalitis virus (TBEV), that cause Lyme borreliosis and tick-borne encephalitis, respectively. Currently there is no vaccine available against Lyme borreliosis but there are human vaccines available against TBEV. Other tick-borne pathogens, which are less prevalent in humans, include *Borrelia miyamotoi*, *Anaplasma phagocytophilum*, *Rickettsia spp*. and *Babesia spp*. The European Center for Disease Prevention and Control has predicted an increase in the incidence of tick-borne diseases, caused by environmental, socio-economic and demographic factors [1, 2]. In the Netherlands, with 17 million inhabitants, it is estimated that there are more than one million tick bites each year [3].

To reduce the risk of human tick-borne diseases, preventive approaches to control tick densities can be achieved by the use of acaricides [4]. However, resistance to acaricides in ticks can occur, and acaricides are harmful for humans, animals and the environment. An alternative approach is an anti-tick vaccine which could lower tick densities as well as pathogen transmission or acquisition [5]. The rationale for the development of a tick-antigen-based vaccine is based on the observation that repeated exposure of certain animal species to tick bites results in the inability of ticks to successfully take a blood meal on these animals, a phenomenon referred to as tick immunity [6]. These animals develop hypersensitivity to ticks after repeated tick bites, and are (partially) protected against tick-borne pathogens [7]. There are currently no vaccines available against *I*. *ricinus* or other *Ixodes* tick species. So far, only one anti-tick vaccine has proven successful in practice, namely a vaccine based on Bm86, which is a glycoprotein located predominantly on the surface of tick midgut cells in *Rhipicephalus microplus* (formerly *Boophilus microplus*) [8, 9]. Bm86 is a concealed antigen to which the host naturally does not develop an immune response, since blood in the gut does not return to the host. A vaccine against Bm86 is available as Gavac and used to be available as TickGARD Plus, but this has recently been withdrawn from the market. Gavac is used in cattle in Latin America and is used to control tick populations by reducing the number of engorging *R*. *microplus* ticks, lowering engorgement weights, and decreasing the number of oviposited eggs [10]. Bm86-based vaccines also protect partially against infection of the parasite *Babesia bovis*. The function of Bm86 is not fully understood, but the vaccine disrupts the tick gut during tick feeding [8]. Others have shown the effect of Bm86-based vaccines on tick feeding in different tick species such as *Rhicephalus annulatus*, *Rhipicephalus decoloratus*, *Hyalomma anatolicum* and *Hyalomma dromedarii*. In contrast, Bm86-based vaccines were not effective against *Amblyomma cajennense*, *Ambylomma variegatum* and *Rhipicephalus appendiculatus* [11–14]. Several studies showed that a vaccine based on Bm86 homologues, such as Haa86 from *H*. *anatolicum* and Hd86 from *Hyalomma scupense*, resulted in lower numbers of engorging ticks after feeding on immunized cattle [15, 16]. To the best of our knowledge, the efficacy of a Bm86-based vaccine against *I*. *ricinus* has never been tested, although such a vaccine could theoretically impair *I*. *ricinus* feeding and transmission of tick-borne pathogens relevant to the human situation, among which *B*. *burgdorferi*. We have recently discovered two conserved Bm86 homologues in *Ixodes* ticks, designated as Ir86-1 and Ir86-2. Quantitative RT-PCR confirmed transcription of both genes specifically in the gut during adult *I*. *ricinus* feeding, suggesting that these proteins are—similar to Bm86—concealed antigens [17]. Ir86-1 (Genbank: GU144602) consists of 619 amino acids, with a predicted molecular weight of 68.0 kDa and is 30% identical and 49% similar to Bm86, whereas Ir86-2 (Genbank: GU979808) consists of 610 amino acids with a predicted molecular weight of 68.4 and is 28% identical and 45% similar to Bm86. Both Ir86-1 and Ir86-2 have a GPI anchor, seven full EGF domains and one partial EGF domain [17]. In addition, a Bm86 homologue was also identified in *Ixodes scapularis* (Genbank: EEC05149), which is an important vector of tick-borne diseases in Northeastern parts of the United States of America [18]. In this study we investigated whether immunizing against recombinant Ir86-1, Ir86-2 or a combination of both in rabbits prevents successful feeding and reproduction of adult *I*. *ricinus*. These three vaccine strategies were tested in two subsequent experiments; in a pilot experiment all three were tested in single rabbits followed by a second experiment in which the most effective vaccine strategy was retested using three rabbits.

## Material and Methods

### Ethics statement

The Animal Care and Use Committee of the University of Amsterdam approved all animal experiments (Permit number: DIX102900). Experiments have been conducted according to national guidelines.

### Animals and ticks

For the immunization six weeks old inbred New Zealand white rabbits (Charles River Laboratories) were used. *I*. *ricinus* adults (Singraven strain, the Netherlands) free from *Borrelia* species, *Anaplasma*, *Ehrlichia*, *Babesia* and *Theileria* were purchased from the Utrecht Centre for Tick-borne Diseases, Utrecht University, the Netherlands. Ticks were maintained at 23°C and 85% relative humidity under a 14 h light, 10 h dark photoperiod.

### Purification of recombinant Ir86-1 and Ir86-2

Ir86-1 (Genbank GU144605.1) and Ir86-2 (GU979808.1) sequences were amplified from *I*. *ricinus* cDNAs using primers Ir86_1_clone_FW CCATGGTCCCCTGTCCTTGGATTG, Ir86_1_clone_RV 5’-CTCGAGCTTTTCCTCGCACAGGTTTC-3’ and 5’Ir86_2_clone_FW CCATGGGTCATCGTCACGTGTTTG-3’ and Ir86_2_clone_RV CTCGAGTCTCTCACAACGTTCTC excluding the signal peptide and GPI anchors regions in both proteins. PCR products were ligated into the pGEM-T easy sequencing vector according to the manufacturer’s instructions (Promega, Madison, WI), transformed in DH5-alpha (Invitrogen, CA, USA) and plated on LB-ampicillin plates. Single colonies were cultured into LB-ampicillin (50 μg/mL) and recombinant DNA was isolated using the Mini-prep KIT (Qiagen, Valencia, CA, USA). Inserts were sequenced using Big Dye Terminator mix, M13 forward or reverse primers, and an automated sequencer (3730 DNA analyzer; all from AB Applied Biosystems, Foster City, CA). From 20 clones, one clone for each protein was selected with the highest similarities to the previous identified sequences (100% for Ir86-1 and 98.8% for Ir86-2). Both sequences were cloned in-frame into the pmt-bip-v5-his tag vector (Invitrogen, CA, USA). Stable transformants were selected with blasticidin, expanded into a 1 L spinner flask and protein expression was induced with copper sulfate and proteins were purified from the supernatant as described by the manufacturer (Invitrogen, CA, USA) using Ni-NTA chromatography cartridges (Qiagen, CA, USA) [19]. The eluted fractions were desalted and concentrated in PBS with a 30 kDa cut-off spin concentrator (Millipore, Billerica, MA). Protein purity was assessed by Coomassie blue on a 7.5% SDS-PAGE gel.

### Detection of native Ir86-1 and Ir86-2 in *I*. *ricinus* gut lysate

Twenty *I*. *ricinus* female ticks were dissected to collect gut tissue and guts were homogenized in 200 uL PBS using a syringe. Tick gut lysate (5 μg) was separated by 10% SDS-PAGE and blotted onto an PVDF-membrane. To detect the presence of native Ir86-1 or Ir86-2, we incubated the PVDF-membrane with antiserum (1:200) from rabbits vaccinated against Ir86-1 or Ir86-2 (see below) and bound antibodies were detected using goat anti-rabbit IgG HRP (1:4000, Cell Signaling, MA). As a control, antiserum from a rabbit vaccinated against ovalbumin was used.

### Study design

Two experiments were performed in both of which four rabbits were immunized and infested with *I*. *ricinus* adult ticks. In a pilot experiment (Experiment A) rabbits were vaccinated with either rIr86-1, rIr86-2, a combination of rIr86-1 and rIr86-2 (from here on referred to as rIr86-combination), or ovalbumin as a control. To validate the findings from experiment A, three animals were vaccinated against rIr86-combination in a second experiment and one rabbit was vaccinated against ovalbumin (Experiment B).

### Vaccine formulations and rabbit immunization

Four different vaccines were used, namely rIr86-1 (100 μg rIr86-1 per immunization) rIr86-2, (100 μg rIr86-2 per immunization) or rIr86-combination (50 μg rIr86-1 and 50 μg rIr86-2). As a control we used ovalbumin (100 μg per immunization, Invitrogen, CA). Recombinant protein in PBS solution was mixed 1:1 with complete Freund’s adjuvant at initial immunization and with incomplete Freund’s adjuvant for boosters. All vaccines were injected subcutaneously, followed by two boosts at 3 and 6 weeks and blood was collected via the ear vein before each vaccine was given. Two weeks after the last booster tick infestation was started (see below). Cut-off for titer was calculated as OD pre-immune serum + 3 SD. Animals were monitored once a day and had continuous access to food and water during the vaccination period. There was no indication for anesthesia or analgesia during the vaccination period.

### ELISA assessment of antibodies in rabbit serum against rIr86-1 and rIr86-2

V5-antibody (Invitrogen, CA, USA) was coated (1 μg/ml in PBS) on high binding microtiter plates (Microlon, Greiner, Germany) overnight at RT. Wells were blocked with PBS/1% BSA at RT for 1 h and incubated with rIr86-1 or rIr86-2 (1 μg/ml) diluted in PBS/ 0.05% Tween20/ 1% BSA for 1 h. Wells were washed and incubated with diluted rabbit serum in PBS/1% BSA and bound antibodies were detected using HRP-conjugated anti-rabbit IgG (Sigma, MO, USA) and TMB as substrate (Sigma-Aldrich, MO, USA). Absorbance at 450 nm–650 nm was detected using the iMark Microplate Reader (Biorad, CA, USA).

### Tick infestation

Two weeks after the final boosters, 25 adult *I*. *ricinus* pairs were placed on each ear (50 *I*. *ricinus* pairs in total per rabbit) in experiment A. To protect the ticks from manipulation by the rabbit, ticks placed on the ears were protected by cotton socks taped to the ears and a neck collar (soft eCollar, MDC exports, UK). In experiment B ticks were placed in an improved feeding chamber, by placing them into a glued capsule on both shaved back flanks, protected by a collar and a medical pet shirt (MPS, the Netherlands). Animals were monitored twice a day during the tick infestation and had continuous access to food and water. There was no indication for anesthesia or analgesia during the tick infestation. After all ticks were collected from the rabbits, serum was collected. Animals were anesthetized with Dexdomitor 0,25 mL / kg and Ketamine (35 mg / kg) after which blood was collected from the cervical artery. After blood collection, Euthasol (pentobarbital 100 mg / kg) injected via the ear vein was used to euthanize the rabbits.

### Tick collection and assessment of tick weight and egg mass

Fully engorged and detached female adult ticks were collected daily from the socks or feeding chamber. Subsequently, ticks were weighted and stored individually in 2,0 mL Eppendorf tubes, which were modified to allow air ventilation. Ticks were assessed for egg mass 6 weeks post feeding.

### Statistical analysis

The significance of the difference between the mean values of the groups was analyzed using Prism 5.0 software (GraphPad Software, San Diego, CA). A one-way analysis of variance (ANOVA) with the Tukey-Kramer multiple-comparison test for multi-group comparisons was used, and p < 0.05 was considered significant.

## Results

### Expression of recombinant Ir86-1 and Ir86-2 and detection of native Ir86-1 and Ir86-2 in *I*. *ricinus* gut lysate

**Fig 1:**
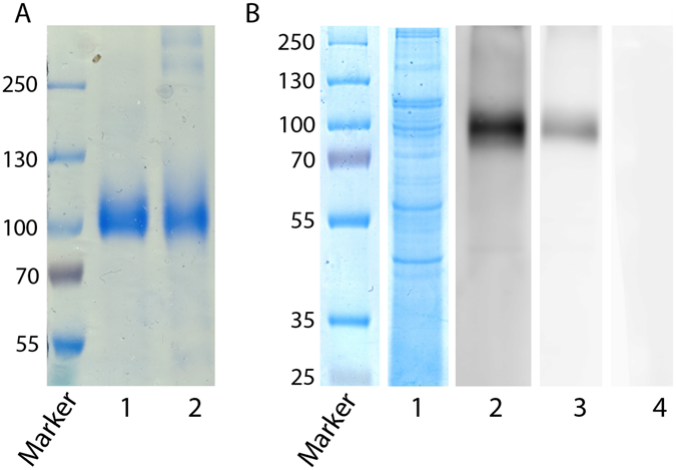
Expression of recombinant Ir86-1 and Ir86-2 and detection of Ir86-1 and Ir86-2 in *I*. *ricinus* gut lysate. **A.** Purified *Drosophila*-expressed recombinant Ir86-1 (Lane 1) and Ir86-2 (Lane 2) electrophoresed on SDS 7.5% polyacrylamide gel and stained with Coomassie blue. **B.** Gut lysate from 20 adult female *I*. *ricinus* tick guts electrophoresed on SDS 10% polyacrylamide gel stained with Coomassie blue (Lane 1) or transferred to a PVDF membrane which was probed with antiserum (1:200) from rabbits vaccinated against rIr86-1 (Lane 2), rIr86-2 (Lane 3) or with antiserum from the control rabbit vaccinated against ovalbumin (Lane 4).

The Ir86-1 and Ir86-2 coding sequences—without the GPI anchor and signal peptide—were cloned and expressed in a *Drosophila* expression system (DES) using the pmt/bip/v5-his vector. The rIr86-1 and rIr86-2 sequence were respectively 100% and 99% identical with the previously identified sequences [17]. After purification, a Coomassie gel showed a molecular size of approximately 90–100 kDa for both proteins (Fig 1A). The presence of native Ir86-1 and Ir86-2 protein in the tick gut was confirmed by an immunoblot in which *I*. *ricinus* tick gut lysate was probed with antiserum from rabbits immunized against rIr86-1 and rIr86-2 (Fig 1B).

### IgG response against rIr86-1 and rIr86-2 in rabbits

**Fig 2:**
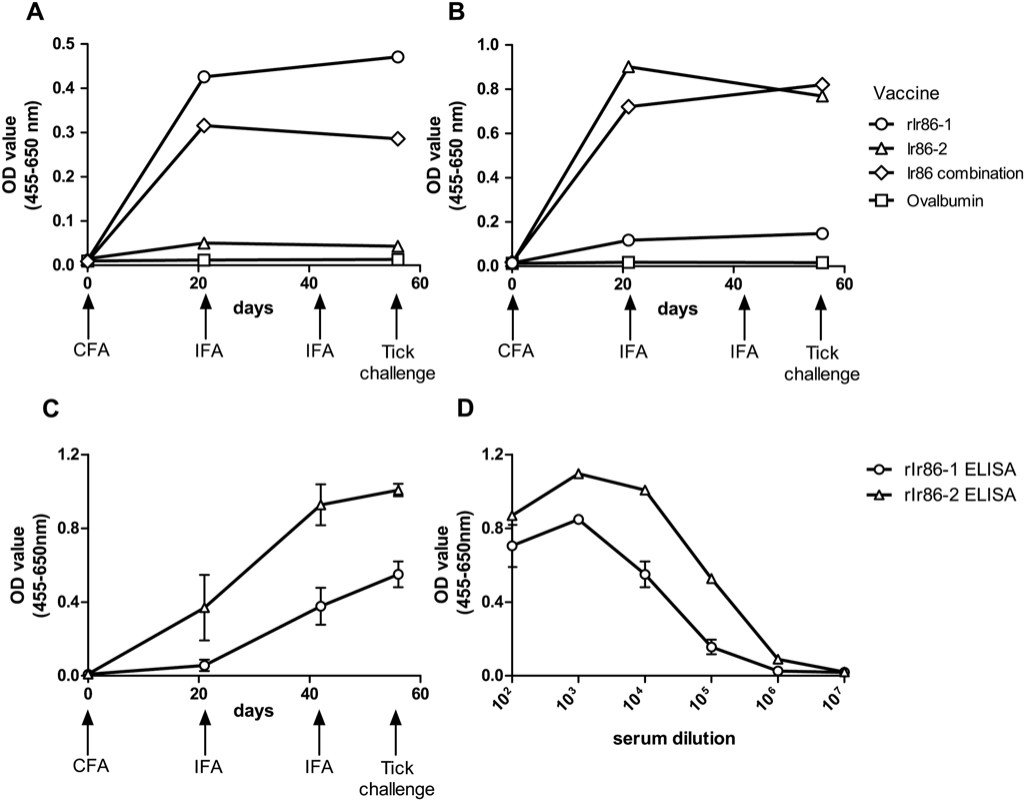
Specificity and IgG response against rIr86-1 and rIr86-2 in experiment A (pilot experiment) and B (confirmatory experiment). IgG in serum (1:10,000) was assessed with ELISA using 1 μg/mL of rIr86-1 and rIr86-2 on anti-V5 coated plates. **A and B**. IgG response in serum from animals in experiment A vaccinated with rIr86-1 (circle), rIr86-2 (triangle), rIr86-combination (diamond) or ovalbumin (square) (n = 1 for each vaccine) reacting to rIr86-1 (A) or rIr86-2 (B). **C.** Mean IgG response in serum (1:10,000) from three animals vaccinated against rIr86-combination in experiment B measuring IgG response to rIr86-1 (circle) or rIr86-2 (triangle). **D.** Mean IgG titer in serum from three animals vaccinated against rIr86-combination in experiment B, diluted 1:10^2^ to 1:10^7^ on ELISA coated with rIr86-1 (circle) or rIr86-2 (triangle). Error bars represent mean ± SEM.

Rabbits were vaccinated in two subsequent experiments, designated as Experiment A (pilot experiment) and B (confirmatory experiment). A sandwich ELISA detecting antibodies against rIr86-1 or rIr86-2 was performed to confirm serum reactivity against both proteins in rabbits. In experiment A, in serum of the rabbit vaccinated against rIr86-1 we detected antibodies against rIr86-1 and antibodies recognizing rIr86-2 were hardly present (Fig 2A and 2B). Similarly, serum from the rIr86-2 vaccinated rabbit reacted with rIr86-2 and only to some extent with rIr86-1 (Fig 2A and 2B). The serum from the rabbit vaccinated against rIr86-combination responded similarly to rIr86-2, but lower to rIr86-1 compared to the single protein vaccinated animals (Fig 2A and 2B). In experiment B, three rabbits were vaccinated against rIr86-combination (Fig 2C). Using an ELISA with a rIr86-1 or rIr86-2 coating, we estimated that at the start of tick feeding (t = 56) the IgG titer in the sera of these three rabbits against rIr86-1 and Ir86-2 was 1:10^5^ and 1:10^6^, respectively (Fig 2D).

### Infestation of *I*. *ricinus* adults on rIr86-immunized or ovalbumin-immunized rabbits

**Fig 3:**
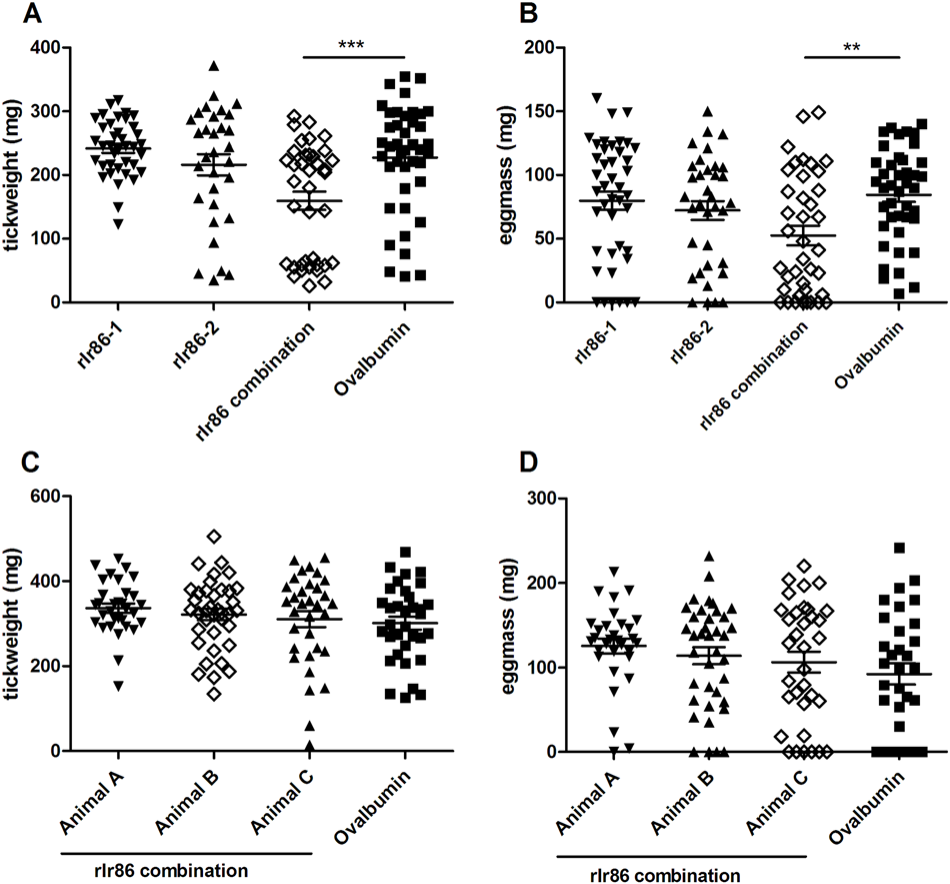
*I*. *ricinus* infestations on rIr86-1, rIr86-2 or rIr86-combination vaccinated rabbits. Rabbits were infested with 50 *I*. *ricinus* adult females and 50 *I*. *ricinus* adult males two weeks after the second boost (t = 56) in a pilot experiment (experiment A) and a confirmatory experiment (experiment B). Each column on the x-axis represents one animal in experiment A (A and B) or experiment B (C and D). **A.** Post feeding female tick weight in experiment A, each dot represents one tick. **B.** Egg mass from experiment A. Fed adult female ticks were stored individually. After 6 weeks, egg mass was weighted. Each dot represent egg mass from one female tick. **C.** Post feeding female tick weight in experiment B. **D.** Egg mass from experiment B. Error bars represent mean ± SEM. Mean values significantly different in a one-way analysis of variance (ANOVA) with the Tukey-Kramer multiple-comparison test for multi-group comparisons are indicated by two asterisk (p < 0.01) or three asterisks (p < 0.001).

Two weeks after the second IFA boost (t = 56 days), the immunized rabbits were infested with 50 female *I*. *ricinus* adults and 50 male *I*. *ricinus* adults. In both experiments, tick repletion and detaching took 7 to 9 days and no differences in numbers of engorged female ticks were seen between the rIr86 vaccinated animals and control animals (ranging from 64–86%). In experiment A, no differences were found between the animals that received rIr86-1 or rIr86-2 vaccination compared to the ovalbumin vaccinated animal (Fig 3A and 3B). However, *I*. *ricinus* female engorgement weights were significantly lower in ticks that had fed on the rIr86 combination-vaccinated rabbit compared to ovalbumin (Fig 3A). No visual abnormalities (i.e. redness in the body) were observed in ticks that had fed on the rIr86-combination rabbit. In line with the reduced engorgement weights, egg weights were also significantly lower from ticks that fed on the rIr86 combination-vaccinated rabbit compared to ovalbumin (Fig 3B). However, these findings were not observed in experiment B, in which three rabbits were immunized against the rIr86 combination and no differences in engorgement weights or egg mass were found (Fig 3C and 3D). Together, the two experiments show that there were no significant effects on tick feeding with any of the three tested vaccines.

## Discussion

Although a vaccine targeting Bm86 or its homologue in the tick gut has proven to be effective against a number of tick species, our study shows that a vaccine based on the Bm86 homologues in *I*. *ricinus* Ir86-1 and Ir86-2 does not prevent successful feeding of adult *I*. *ricinus*. We were able to successfully immunize rabbits against rIr86-1 and rIr86-2 with low cross reactivity of the antibodies and both proteins could be detected in the lysate of the guts from adult *I*. *ricinus* ticks. We chose CFA and IFA as vaccine adjuvants to ensure high IgG antibody development. Using vaccines with both rIr86-1 and rIr86-2 resulted in a higher IgG response in serum against rIr86-2 compared to rIr86-1, but the titer for both antigens was similar or even higher compared to other vaccination studies [20, 21]. To study the effect of the vaccine on tick feeding, our study was performed in a two-step approach. First, three different vaccine designs were tested in a single rabbit after which the most effective vaccine was validated using three rabbits to confirm our initial findings. Although vaccination with a combination of rIr86-1 and rIr86-2 resulted in lower tick engorgement weights and egg mass in the pilot experiment, no differences were found in the second confirmatory experiment. The initial findings in the pilot experiment could be due to variance in animals or ticks. The same batch of purified recombinant proteins and ticks from the same tick colony were used in both experiments. In addition, the IgG levels in serum against rIr86-1 and rIr86-2 were similar in both experiments. We observed a higher average tick weight in the second experiment compared to the first, e.g. 307 mg (σ = 89.9) versus 227 mg (σ = 80.9) when comparing ticks that fed on the control vaccine rabbits. The only difference in experimental procedures between both experiments is that in experiment A ticks were placed on the ears of the rabbits with a sock and collar as protection, while in experiment B ticks were placed in plastic feeding chambers on the flanks to improve protection from manipulation by the rabbits. In experiment A the rabbit receiving the rIr86-combination might have experienced more severe itching at the tick bite site (the ear), and more opportunity than the rabbits in experiment B to remove the ticks from the skin by itself. Although this might have been a reason for the observed increased overall tick weights, it does not explain the difference in phenotype between the two experiments.

We only studied the effect of this vaccine in rabbits and can therefore not predict whether such a vaccine would be effective in other mammalian animal species. A possible explanation as to why the rIr86 vaccine was not effective against *I*. *ricinus* in our model could be that the lifecycle of *I*. *ricinus* is different from *R*. *microplus*. *R*. *microplus* is a one-host tick, which means that ticks feed on one host during their life and ticks are exposed to serum antibodies from the same individual at the larval, nymphal and adult life stage before laying eggs. *I*. *ricinus* is a three host tick and in our study, ticks were only exposed to rIr86 antibodies at the adult feeding stage. A repeated or prolonged exposure could be crucial for the efficacy of a vaccine against Bm86 or Bm86-homologues. In addition, although CFA/IFA is a robust way of vaccinating and we did observe high antibody titers, other vaccine formulations might also be worth investigating.

Bm86 based vaccines are the only available commercial anti-tick vaccine and have showed protection against several tick species. Although we did not see any effect, other studies have reported other *I*. *ricinus* antigens as possible vaccine candidates to impact *I*. *ricinus* tick feeding or pathogen transmission. Hajdusek et al. recently showed that the tick protein ferritin 2 in rabbit vaccination experiments lowered *I*. *ricinus* engorgements weights and reduced egg mass, making it a potential candidate for an anti-*I*. *ricinus* vaccine [20]. Furthermore, a truncated form of 64P has also been identified as a candidate for an anti-tick vaccine to prevent TBEV transmission [21]. Finally, vaccination experiments in mice using *I*. *scapularis* salivary gland proteins TSLPI, Salp15 and tHRF were effective in lowering *B*. *burgdorferi* transmission [22–24]. However, none of the known candidates have made it to the market as an anti-tick vaccine targeting *Ixodes* ticks. Future research should focus on the discovery of new vaccination candidates, vaccine combinations and vaccination strategies against *I*. *ricinus*. Recently, a grant was awarded to ANTIDotE, a consortium of seven European partners, which aims to deliver vaccine candidates against *I*. *ricinus* and will explore how such vaccines could be implemented in European health systems [2].

In conclusion, we successfully established antibody production against recombinant *I*. *ricinus* Bm86 homologues Ir86-1 and Ir86-2 in rabbits. However, using CFA and IFA, vaccinating rabbits against rIr86-1, rIr86-2 or both did not interfere with adult tick feeding or egg production.

## Supporting Information

S1 Table Tick weights and egg mass from both vaccination experiments. (PDF)

## References

[1] Lindgren E, Andersson Y, Suk JE, Sudre B, Semenza JC. Public health. Monitoring EU emerging infectious disease risk due to climate change. Science. 2012;336: 418–9. 10.1126/science.1215735 22539705

[2] Sprong H, Trentelman J, Seemann I, Grubhoffer L, Rego RO, Hajdusek O, et al ANTIDotE: anti-tick vaccines to prevent tick-borne diseases in Europe. Parasites & vectors. 2014;7: 77. 24559082 10.1186/1756-3305-7-77 PMC3933510

[3] Hofhuis A, Harms MG, Giessen JWBvd, Sprong H, Notermans DW, Pelt Wv. Ziekte van Lyme in Nederland 1994–2009: Aantal huisartsconsulten blijft toenemen. Is voorlichting en curatief beleid genoeg?. Infectieziekten Bulletin Jaargang 21 nummer 03 2010;21: 84–7.

[4] Pages F, Dautel H, Duvallet G, Kahl O, de Gentile L, Boulanger N. Tick repellents for human use: prevention of tick bites and tick-borne diseases. Vector Borne Zoonotic Dis. 2014;14: 85–93. 10.1089/vbz.2013.1410 24410143

[5] Schuijt TJ, Hovius JW, van der Poll T, van Dam AP, Fikrig E. Lyme borreliosis vaccination: the facts, the challenge, the future. Trends Parasitol. 2011;27: 40–7. 10.1016/j.pt.2010.06.006 20594913

[6] Trager W. Acquired immunity to ticks. J Parasitol. 1939;7: 15–20.

[7] Narasimhan S, Deponte K, Marcantonio N, Liang X, Royce TE, Nelson KF, et al Immunity against *Ixodes scapularis* salivary proteins expressed within 24 hours of attachment thwarts tick feeding and impairs *Borrelia* transmission. PLoS One. 2007;2: e451 17505544 10.1371/journal.pone.0000451 PMC1866177

[8] Willadsen P, Riding GA, McKenna RV, Kemp DH, Tellam RL, Nielsen JN, et al Immunologic control of a parasitic arthropod. Identification of a protective antigen from *Boophilus microplus* . J Immunol. 1989;143: 1346–51. 2745982

[9] Gough JM, Kemp DH. Localization of a low abundance membrane protein (Bm86) on the gut cells of the cattle tick *Boophilus microplus* by immunogold labeling. J Parasitol. 1993;79: 900–7. 8277383

[10] de la Fuente J, Almazan C, Canales M, Perez de la Lastra JM, Kocan KM, Willadsen P. A ten-year review of commercial vaccine performance for control of tick infestations on cattle. Animal health research reviews / Conference of Research Workers in Animal Diseases. 2007;8: 23–8. 17692140 10.1017/S1466252307001193

[11] Canales M, Almazan C, Naranjo V, Jongejan F, de la Fuente J. Vaccination with recombinant *Boophilus annulatus* Bm86 ortholog protein, Ba86, protects cattle against *B*. *annulatus* and *B*. *microplus* infestations. BMC Biotechnol. 2009;9: 29 10.1186/1472-6750-9-29 19335900 PMC2667501

[12] de Vos S, Zeinstra L, Taoufik O, Willadsen P, Jongejan F. Evidence for the utility of the Bm86 antigen from *Boophilus microplus* in vaccination against other tick species. Exp Appl Acarol. 2001;25: 245–61. 11523920 10.1023/a:1010609007009

[13] Rodriguez-Valle M, Taoufik A, Valdes M, Montero C, Ibrahin H, Hassan SM, et al Efficacy of *Rhipicephalus (Boophilus) microplus* Bm86 against *Hyalomma dromedarii* and *Amblyomma cajennense* tick infestations in camels and cattle. Vaccine. 2012;30: 3453–8. 10.1016/j.vaccine.2012.03.020 22446633

[14] Odongo D, Kamau L, Skilton R, Mwaura S, Nitsch C, Musoke A, et al Vaccination of cattle with TickGARD induces cross-reactive antibodies binding to conserved linear peptides of Bm86 homologues in *Boophilus decoloratus* . Vaccine. 2007;25: 1287–96. 17070625 10.1016/j.vaccine.2006.09.085

[15] Azhahianambi P, De La Fuente J, Suryanarayana VV, Ghosh S. Cloning, expression and immunoprotective efficacy of rHaa86, the homologue of the Bm86 tick vaccine antigen, from *Hyalomma anatolicum anatolicum* . Parasite immunology. 2009;31: 111–22. 10.1111/j.1365-3024.2008.01082.x 19222782

[16] Galai Y, Canales M, Ben Said M, Gharbi M, Mhadhbi M, Jedidi M, et al Efficacy of *Hyalomma scupense* (Hd86) antigen against *Hyalomma excavatum* and *H*. *scupense* tick infestations in cattle. Vaccine. 2012;30: 7084–9. 10.1016/j.vaccine.2012.09.051 23036501

[17] Nijhof AM, Balk JA, Postigo M, Rhebergen AM, Taoufik A, Jongejan F. Bm86 homologues and novel ATAQ proteins with multiple epidermal growth factor (EGF)-like domains from hard and soft ticks. Int J Parasitol. 2010;40: 1587–97. 10.1016/j.ijpara.2010.06.003 20647015 PMC2998001

[18] de la Fuente J, Estrada-Pena A, Venzal JM, Kocan KM, Sonenshine DE. Overview: Ticks as vectors of pathogens that cause disease in humans and animals. Front Biosci. 2008;13: 6938–46. 18508706 10.2741/3200

[19] Schuijt TJ, Narasimhan S, Daffre S, DePonte K, Hovius JW, Van't Veer C, et al Identification and characterization of *Ixodes scapularis* antigens that elicit tick immunity using yeast surface display. PLoS One. 2011;6: e15926 10.1371/journal.pone.0015926 21246036 PMC3016337

[20] Hajdusek O, Almazan C, Loosova G, Villar M, Canales M, Grubhoffer L, et al Characterization of ferritin 2 for the control of tick infestations. Vaccine. 2010;28: 2993–8. 10.1016/j.vaccine.2010.02.008 20171306

[21] Labuda M, Trimnell AR, Lickova M, Kazimirova M, Davies GM, Lissina O, et al An antivector vaccine protects against a lethal vector-borne pathogen. PLoS Pathog. 2006;2: e27 16604154 10.1371/journal.ppat.0020027 PMC1424664

[22] Dai J, Wang P, Adusumilli S, Booth CJ, Narasimhan S, Anguita J, et al Antibodies against a tick protein, Salp15, protect mice from the Lyme disease agent. Cell Host Microbe. 2009;6: 482–92. 10.1016/j.chom.2009.10.006 19917502 PMC2843562

[23] Schuijt TJ, Coumou J, Narasimhan S, Dai J, Deponte K, Wouters D, et al A tick mannose-binding lectin inhibitor interferes with the vertebrate complement cascade to enhance transmission of the lyme disease agent. Cell Host Microbe. 2011;10: 136–46. 10.1016/j.chom.2011.06.010 21843870 PMC3170916

[24] Dai J, Narasimhan S, Zhang L, Liu L, Wang P, Fikrig E. Tick histamine release factor is critical for *Ixodes scapularis* engorgement and transmission of the lyme disease agent. PLoS Pathog. 2010;6: e1001205 10.1371/journal.ppat.1001205 21124826 PMC2991271

