# Supplementary material for: Vaccination against Bm86 Homologues in Rabbits Does Not Impair Ixodes ricinus Feeding or Oviposition
Source: PLoS One. 2015 Apr 28;10(4):e0123495. doi: 10.1371/journal.pone.0123495 (PMC4412674; doi:10.1371/journal.pone.0123495)
Supplement: S1 Table — (PDF) [file pone.0123495.s001.pdf]

Experiment A. Tickweights (in mg)

Tick	rIr86-1	rIr86-2	rIr86 combination	Ovalbumin
1	185	221	60	249
2	317	312	50	213
3	248	49	59	148
4	253	35	50	221
5	204	307	58	41
6	244	324	223	219
7	245	43	218	240
8	196	94	292	293
9	232	244	208	104
10	289	270	283	148
11	233	45	231	299
12	297	179	257	43
13	274	204	55	76
14	311	196	59	239
15	293	372	69	90
16	223	292	207	245
17	280	271	55	213
18	275	164	64	190
19	214	287	218	236
20	220	214	237	251
21	239	228	229	230
22	192	126	180	309
23	245	265	190	299
24	295	266	142	352
25	202	274	223	237
26	291	298	26	249
27	246	132	32	343
28	201	154	41	329
29	216	236	261	296
30	288	302	216	276
31	260	295	62	300
32	216		232	248
33	210		254	283
34	122		204	278
35	256		238	266
36	149		144	355
37	266		151	222
38	263		279	275
39				126
40				298
41				48
42				179

Experiment A. Eggmass (in mg)

Tick	rIr86-1	rIr86-2	rIr86 combination	Ovalbumin
1	90	107	26	137
2	149	107	5	100
3	118	23	34	60
4	111	29	15	108
5	103	13	24	12
6	97	112	109	78
7	0	150	110	125
8	74	0	146	134
9	44	0	112	19
10	0	99	70	76
11	129	77	122	67
12	121	23	149	23
13	113	104	6	44
14	120	83	10	72
15	148	78	27	39
16	108	98	67	123
17	124	106	20	99
18	126	134	10	101
19	125	74	104	55
20	100	83	111	134
21	110	100	88	95
22	91	132	48	67
23	126	75	0	132
24	160	100	0	90
25	40	71	82	100
26	38	72	0	68
27	0	125	0	66
28	84	19	0	26
29	71	47	0	114
30	80	88	41	75
31	123	45	23	133
32	24		77	92
33	34		0	110
34	23		99	95
35	68		103	110
36	0		67	140
37	0		56	112
38	81		87	87
39				39
40				102
41				7
42				90

Experiment B. Tickweights (in mg)

Tick	Animal A	Animal B	Animal C	Ovalbumin
1	308	332	384	349
2	410	416	148	433
3	312	376	352	271
4	293	441	347	323
5	371	325	376	338
6	290	320	455	267
7	334	236	366	270
8	431	330	344	383
9	437	355	289	334
10	311	380	243	207
11	348	331	386	341
12	329	444	328	469
13	347	372	407	417
14	324	379	362	293
15	403	324	354	396
16	367	286	60	133
17	275	311	14	400
18	302	366	425	135
19	320	505	393	422
20	403	351	186	363
21	453	181	347	331
22	350	398	402	282
23	285	339	434	277
24	152	383	323	249
25	344	309	220	147
26	346	420	420	337
27	364	279	277	377
28	293	295	449	215
29	344	206	235	228
30	303	173	317	213
31	416	333	224	345
32	213	187	242	275
33		377	143	126
34		134		298
35		255		
36		249		
37		326		
38		205		

Experiment B. Eggmass (in mg)

Tick	Animal A	Animal B	Animal C	Ovalbumin
1	119	160	152	98
2	149	176	67	203
3	135	171	167	115
4	119	208	0	131
5	113	147	169	61
6	129	146	204	114
7	133	77	156	0
8	183	105	84	180
9	213	170	171	152
10	131	167	129	75
11	132	147	18	125
12	138	168	165	242
13	146	160	157	194
14	151	181	200	0
15	191	143	160	172
16	164	138	139	0
17	95	138	0	0
18	121	119	0	30
19	128	232	220	180
20	190	61	0	159
21	152	35	0	121
22	156	138	168	0
23	134	118	188	105
24	23	179	197	65
25	87	158	124	0
26	127	81	70	100
27	144	110	135	61
28	0	51	79	143
29	113	140	60	0
30	4	0	57	53
31	71	87	98	0
32	98	71	65	79
33		0	0	88
34		59		71
35		54		
36		0		
37		0		
38		41		
